# Backcrossing Failure between Sikitita Olive and Its Male Parent Arbequina: Implications for the Self-Incompatibility System and Pollination Designs of Olive Orchards

**DOI:** 10.3390/plants13202872

**Published:** 2024-10-14

**Authors:** Julián Cuevas, Fernando M. Chiamolera, Virginia Pinillos, Francisco Rodríguez, Irene Salinas, Diego Cabello, Alenka Baruca Arbeiter, Dunja Bandelj, Marina Raboteg Božiković, Gabriela Vuletin Selak

**Affiliations:** 1 Department of Agronomy, University of Almería, Campus de Excelencia Internacional Agroalimentario (ceiA3), 04120 Almería, Spain; fmc1984@ual.es (F.M.C.); vpinillo@ual.es (V.P.); fra460@ual.es (F.R.); isr122@ual.es (I.S.); 2 Department of Agronomy, University of Córdoba, Campus de Excelencia Internacional Agroalimentario (ceiA3), 14071 Córdoba, Spain; cabello@uco.es; 3 Faculty of Mathematics, Natural Sciences and Information Technologies, University of Primorska, SI-6000 Koper, Slovenia; alenka.arbeiter@upr.si (A.B.A.); dunja.bandelj@upr.si (D.B.); 4 Institute for Adriatic Crops and Karst Reclamation, 21000 Split, Croatia; marina.raboteg@krs.hr (M.R.B.); gabriela.vuletin.selak@krs.hr (G.V.S.)

**Keywords:** *Olea europaea*, gametophytic self-incompatibility, sporophytic self-incompatibility, pollination groups, pollinizer selection, pollen–pistil interactions, fruit set, seed paternity

## Abstract

Backcrossing between Sikitita and its male parent Arbequina, offers the possibility to check the suitability of different self-incompatibility models proposed for olive. To determine Sikitita’s response to self- and cross-pollination treatments, including pollination with its father Arbequina, we compared the parameters following pollen–pistil interaction, the resulting initial and final fruit set, and the paternity of the seeds produced under different crosses. The results showed that Sikitita behaves as a self-incompatible cultivar due to the inhibition of pollen tube growth in the pistil of self-pollinated flowers. This incompatibility reaction led to a significant reduction of self-fertilization and fruit set. Seed paternity analyses confirmed the self-incompatibility response of Sikitita. A similar incompatibility response was observed in Sikitita flowers when hand-pollinated with pollen of Arbequina and Koroneiki. On the contrary, cross-pollination with Arbosana gave excellent results, with analyses showing that pollen of Arbosana is largely preferred by Sikitita to father its seeds more than the pollen of other cultivars presented in the orchard. The backcross failure of Sikitita with Arbequina pollen suggests that the self-incompatibility system in olives is not of the gametophytic type. In contrast, pollination tests fit features of previously reported sporophytic self-incompatibility systems. However, some amendments are proposed, among them the incompatibility groups for Sikitita and Koroneiki.

## 1. Introduction

The domesticated olive tree (*Olea europaea* subsp. *europapea* L.) is a preferential allogamous, partially self-incompatible species [1], as the mere condition of prevalent wind pollination in the crop may suggest. The olive self-incompatibility response is characterized by the inhibition of pollen tube growth in the stigma and in the style of self-pollinated flowers [2,3,4,5]. The reduced and delayed pollen tube growth leads to lower levels of self-fertilization and then, quite often, to significant reductions in fruit set and yield under self-pollination conditions [6,7,8,9].

However, despite the clear characterization of the olive self-incompatibility reaction, there is not yet a unanimous consensus about the system of self-incompatibility operating in olive. Self-incompatibility (SI) is a widespread mechanism to promote outcrossing in angiosperms based on the recognition and rejection of self-pollen to achieve ovule fertilization. Two different systems operate in angiosperms: gametophytic self-incompatibility (GSI), frequently based on the activity of RNases in the style, enzymes that penetrate pollen tubes formed from self-pollen and trigger a cascade of events leading to programmed cell death that reduces the chances for self-fertilization. The second system is the sporophytic self-incompatibility (SSI), a system in which, with some exceptions, the expression of the SRK protein kinase in the stigma leads also to programmed cell death and to the blockage of the germination of self-pollenation [10].

In the GSI system, each pollen grain possesses one single S-allele, and its phenotype corresponds to this single S-allele. In contrast, in the well-studied SSI of the families Brassicaceae and Asteraceae, the male determinants of the SI reaction are expressed earlier in pollen development, when the grains are still in the diploid anther, and, therefore, the exine of all pollen grains is coated by both S-determinants. This makes haploid pollen grains behave with a diploid S-phenotype, coinciding with the genotype of the male sporophyte (this is where the term sporophytic SI comes from). In SSI, if a pollen grain lands on a stigma expressing at least one of the two determinants, the incompatibility reaction is triggered and the pollen grain does not germinate or, if it germinates, is arrested shortly after [11,12,13].

Some authors [2,4,10,14] have stated that olive possesses a GSI, mainly because the pollen–pistil interaction process more clearly affected to avoid or limit self-fertilization is the blockage of the growth of the pollen tubes in the transmitting tissue of the pistil. However, the S-alleles in olive have not been identified, despite the fact that RNase activity has been detected at the stigma surface and in pollen tubes growing in the pistil of olive flowers [15]. Some other authors [16,17,18] defend that the SI operating in olive is of the SSI system. In strong support of this hypothesis is the fact that in the family Oleaceae, SSI has been found to operate in *Fraxinus* [19], *Phillyrea* [20], and *Ligustrum* [21], and the system of SI rarely differs among genera of the same family [22]. Although SI has evolved independently on many occasions in different families, only a few of them contain both SI systems.

**Figure 1:**
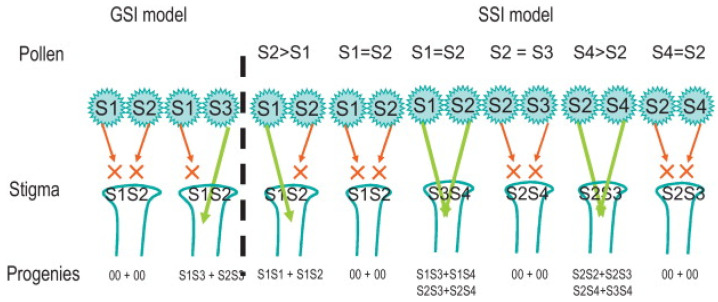
Gametophytic (GSI) and sporophytic self-incompatibility (SSI) models as proposed by Breton and Bervillé in [16]. Published by Elsevier Masson SAS. All rights reserved. X means pollen rejection; the arrow means pollen acceptance; > means dominant upon; 00 means no possible progenies.

Collani et al. [17] proposed that olive expresses a SSI typical of the family Brassicaceae. Their proposal is based on the fact that the female SSI determinants (S-locus receptor kinase and S-locus glycoprotein) are more abundant in female organs than in the anthers, and that the male SSI determinant (S-locus cysteine-rich protein) has a peak in the anthers before anthesis. On the contrary, these authors did not find any evidence for S-RNase and the genes responsible for GSI. In the assignment of SSI to olive, Breton and Bervillé [16] introduced a variant over this classic view of SSI and defended the idea that SSI in olive is based on the dominance of some S-alleles over others. In this variant of SSI, reported more than 70 years ago in wild guayule (*Parthenium argentatum*), a species from the Asteraceae family [23], Breton and Bervillé [16] postulated a SSI system initially with five alleles; these same authors identified them in many French olive cultivars (and in some others from Spain) and selected the best pollinizers for them. Some years later, Farinelli et al. [24] added a sixth S-allele and extended previous work by including main Italian cultivars (see Figure 1 for a complete explanation of the different alternatives for the SI system in olive).

More recently, Saumitou-Laprade et al. [18] analyzed the pollen–pistil interaction 16 h after pollination on a small sample of flowers of several olive genotypes and confirmed that the SI reaction is widely distributed among olive cultivars and mainly expressed as limited pollen tube growth in the pistils of self-pollinated flowers. However, these authors defend that olive exhibits a homomorphic diallelic SSI (DSI) and grouped many olive genotypes, identified using codes, in only two groups (G1 and G2), with genotypes S1S2 and S1S1, respectively. They proposed that the genotypes within a group are inter-incompatible, but compatible with the genotypes of the second group. In their model, they add a dominance relationship, with S2 dominant over S1. The authors have later extended their work and classified many olive cultivars, now fully identified, in these two groups [25] and reported the same DSI system operating in the closed related form, *Olea europaea* subsp. *laperrinei* [26]. According to this theory, roughly half of the cultivars must belong to each group, and so the cases of inter-incompatibility reactions should be common. However, there are many fewer cases of inter-incompatible crosses reported in olive field studies [27,28,29].

Recent olive breeding programs and the release of new cultivars offer us a chance to test the SI system in the species, crossing the new cultivars with their parents. In this regard, if the SI in olive is GSI, in backcrosses, half of the pollen grains, those carrying out the same S-allele, would be blocked after germination. In contrast, the other half of pollen grains, those with no coincident S-alleles, would grow in the style without any impediment and fertilize the ovule in the same proportion, more or less, as in crosses where no S-alleles coincide. In other words, backcrossing will produce the similar fruit set and same yield than crosses between unrelated genotypes. On the contrary, if the SI system in olive is SSI and of the type operating in Brassicaceae and Asteraceae, we may expect the rejection of all pollen grains, and then the inter-incompatibility reaction in the backcross will produce a much-reduced fruit set, classifying the cross as inter-incompatible.

With the aim of further deepening in the knowledge of SI in olive and determining Sikitita incompatibility behavior, we pollinated the flowers of Sikitita olive cultivar with pollen grains of its father Arbequina, and compared pollen–pistil interaction parameters, initial and final fruit set, and determined also the paternity of the seeds in two consecutive seasons. Thus, we have compared self-pollination with open-pollination and with the backcross Sikitita × Arbequina, and with cross-pollination treatments with supposedly unrelated cultivars Arbosana and Koroneiki, the latter tested only in the second year.

## 2. Results

### 2.1. Initial and Final Fruit Set

Sikitita behaved as a strongly self-incompatible genotype, producing very few fruits under self-pollination, almost none in 2022, and a little more in 2023 (Table 1). Open pollination, on the contrary, produced the highest initial and final fruit set in both seasons, while cross-pollination with Arbosana pollen gave very close values to the optimal treatment represented by the unrestricted and continuous open-pollination. In an intermediate position, Arbequina pollen (the father of Sikitita) significantly increased initial and final fruit set in season 2022, but not in season 2023, when the differences with respect to self-pollination did not reach statistical significance. On the other hand, cross-pollination using Koroneiki significantly increased initial and final fruit set compared to self-pollination (Table 1), but less than open- and cross-pollination with Arbosana pollen did (Table 1).

The index of self-incompatibility (ISI) fluctuated between 0.07 and 0.11 in season 2022, illustrating the strong SI response of Sikitita in this season. In 2023, ISI values were in the range between 0.18 for open-pollination and 0.44 when compared to the final fruit set obtained under cross-pollination with Arbequina pollen (Table 1).

### 2.2. Pollen–Pistil Interaction

Pollen adhesion was high in all treatments in both seasons, with flowers adhering more than 300 pollen grains per stigma in most treatments, with the only exceptions of open-pollination in 2022 and cross-pollination with Arbequina pollen in 2023, which had fewer pollen grains adhered on the stigmas on average (Table 2). In contrast, the percentages of pollen germination calculated by dividing the number of germinated pollen grains by the number of adhered pollen grains were low, with percentages between 17 and 18% in 2022, a little less for cross-pollination with Arbequina pollen (14%). The percentage of pollen germination was even lower in 2023, with values between 9 and 12%, except under open-pollination treatment, which reached an average value of 15%.

Higher differences among pollination treatments were found in pollen tube growth. In this regard, cross-pollination using Arbosana pollen stands out, reaching the highest values both seasons. Open-pollination did provide good pollen tube growth in 2022 as well as in 2023, although the growth of the pollen tubes in the pistils started a bit later, and massive pollen tube growth was only seen in flowers sampled 4 and 8 days after pollination. In contrast to these two treatments, self-pollination failed to produce substantial pollen tube growth, showing a poor performance of self-pollen in this regard in both seasons, reaching values of only 0.07 and 0.15 on the scale from 0 to 3 (Table 3). The same failure in pollen tube growth can be attributed to the cross-pollination treatment using Arbequina pollen, especially in 2023. Reduced pollen tube growth was also observed in flowers pollinated with Koroneiki (pollen tube growth in the scale 0–3 = 0.25), although this cultivar was tested as pollinizer only in 2023 (Table 3).

The inhibition of pollen tube growth found in self- and cross-pollinated flowers with pollen of Arbequina (as well as with pollen of Koroneiki) led to a more significant and important reductions of fertilization in both seasons, when compared to the most successful cross-pollination with Arbosana pollen (overall chi square test = 20.20; *p* = 0.0002 in 2022; and overall chi square test = 36.42; *p* < 0.0001 in 2023). Open-pollination showed intermediate values for ovule fertilization, but it also significantly increased the levels of fertilization with regard to self-pollination. However, the differences did not reach statistical significance with respect to other cross-pollination treatments (Table 3).

### 2.3. Seed Paternity Analyses

In 2022, the paternity of the seeds was clearly assigned to 141 out of the 184 embryos sampled (Table 4). On the contrary, the LOD scores obtained did not allow us to establish clear paternity in the remaining 43 embryos, with the level of certainty fixed for our analysis (LOD values below 0.40). These disputed cases are not considered in the discussion of the results. However, the tentative assignment of the most likely paternity in those cases (Supplementary Table S1) does not differ from the distribution of seed paternity assigned when the LOD score was above the threshold value (overall chi square in open-pollination = 4.72; *p* = 0.19).

Seed paternity analyses showed that, in 2022, some treatments had abundant pollen contamination. Thus, while cross-pollination with Arbosana pollen yielded almost all embryos (46 out of 47 embryos) fathered by Arbosana itself, in the case of self-pollination roughly half of the seeds were, in fact, fathered by its own Sikitita pollen (the other half, except one, was fathered by Arbosana) (Table 4). Pollen contamination was even more pronounced in cross-pollination using Arbequina pollen. In this treatment, the majority of the seeds (37 out of 55) were undoubtedly fathered by the pollen of Arbosana instead of by the hand-applied pollen of Arbequina, while only 9 out of 55 seeds were in fact the results of cross-fertilization with Arbequina pollen, suggesting the lack of affinity between Sikitita and its male parent (Table 4). Furthermore, in nine seeds of this treatment, where the LOD score did not allow the establishment of paternity for sure, the embryos were more likely fathered also by Arbosana (Supplementary Table S1). The success of Arbosana pollen on Sikitita flowers was repeated when we analyzed the genotypes that fathered the embryos in open-pollinated flowers. In this treatment, where unrestricted and continuous arrival of pollen from different cultivars happened, Arbosana pollen was responsible for the production of 26 out of 37 seeds, while Arbequina fathered only six seeds, Tosca four, and Koroneiki just one seed (Table 4).

Given the high level of confidence obtained when Arbosana pollen was applied to the flowers of Sikitita, we did not repeat the analyses of seed-paternity in this cross-pollination treatment in 2023. On the contrary, we focused on repeating the analyses of seed-paternity in cross-pollination with pollen of Arbequina, given its poor performance the previous year. Seed-paternity analyses in 2023 also included cross-pollination with Koroneiki pollen. In this second season, we analyzed 163 seeds, from which the paternal genotype that achieved fertilization was clearly established for 129 seeds. In the remaining 34 seeds, LOD score values were below the threshold (0.89), so their likely paternity is not further discussed, although the data are presented in Supplementary Table S2.

The results of season 2023 obtained from seeds in which paternity was established confirmed the strong affinity between Sikitita flowers and Arbosana pollen. In this second season, Arbosana fathered 37 out 61 embryos obtained from the seeds of open-pollination treatment, while Koroneiki fathered only nine seeds, Arbequina fathered eight seeds and Tosca the remaining seven seeds (Table 5).

On the other hand, and similarly to what was observed in 2022, only one seed was the product of self-fertilization in the fruit collected from the bagged shoots that constitute the self-pollination treatment, highlighting the strong SI response of Sikitita. Again, as occurred in 2022, Arbosana fathered most of the seeds in this treatment (28 out of 38), followed by Tosca (8 out of 38 seeds), while Koroneiki fathered only one seed (Table 5).

Even more surprising, but confirming the results of 2022, it was that, in the cross-pollination treatment with Arbequina pollen, Arbosana fathered again more embryos (5) than Arbequina pollen (2) that was copiously applied by hand (Table 5). In this treatment and season, Tosca pollen was able to fertilize six seeds and Koroneiki only one, again underlining the good affinity of the pollen of Arbosana and Tosca with Sikitita flowers and the poor performance of Arbequina and Koroneiki pollen fertilizing Sikitita flowers. Actually, the poor behavior of Koroneiki pollen on Sikitita flowers was repeated in the treatment of cross-pollination with pollen of this cultivar, where Koroneiki was only able to father 1 out of the 16 seeds in the samples where LOD scores were above the threshold value. In this treatment, Arbosana pollen was again the most successful pollen donor, being responsible for the production of eight seeds (out of sixteen), closely followed by Tosca (six out of sixteen), and finally Arbequina, fathering only one seed (Table 5).

## 3. Discussion

### 3.1. Self-Incompatibility Reaction in Sikitita

Sikitita behaved as a strongly self-incompatible cultivar with a very reduced initial and final fruit set under self-pollination compared to open- and cross-pollination with Arbosana pollen. In this regard, the ISI of Sikitita was around or below 0.10 in season 2022 and around 0.20 in 2023 in these two treatments (Table 1). SI has been repeatedly reported in many olive genotypes, but it is important to confirm this behavior, especially in newly released cultivars intended for very large, high-density monovarietal orchards, where pollination deficits may likely appear.

The SI reaction in Sikitita was characterized by the inhibition of pollen tube growth in the stigma and in the style of self-pollinated flowers, despite good levels of pollen adhesion and germination in the first days after pollination. This syndrome, first reported more than 50 years ago by Bradley and Griggs [2] on Manzanillo (syn. Manzanilla de Sevilla) olive, has been frequently observed not only in olive but also in other genera of the family Oleaceae [18,21,30]. However, because the incompatibility reaction occurs after germination, the interpretation has led to controversy in the context of SI systems because of the timing and place of self-pollen rejection. Since pollen germination on the stigma surface is seldom and limitedly affected by self-pollination treatment in olive, early interpretations suggested that this species possesses a GSI [2]. Binucleate mature pollen grains and wet stigmas, as olive has [4], gave additional support to this interpretation [14], although the correlation between those characteristics and GSI has numerous exceptions [31] and, therefore, the assignment of GSI for only these traits should not be performed.

Our results did show some occasional differences in pollen adhesion among treatments, but those differences were not critical and are interpreted as a consequence of the procedures followed to perform pollination in the different treatments. In this regard, pollen adhesion in open-pollination treatment relied on wind transport, while in cross-pollination we applied a copious amount of pollen grains by hand. This procedure favored pollen adhesion in cross-pollination treatments, although the differences among treatments were not very important (Table 2). Pollen adhesion under self-pollination was somehow favored by the frequent shaking of the pollination bags by hand and by the wind, but the amount of pollen is limited to the grains formed in the bagged flowers. Nonetheless, self-pollination treatment also showed a large amount of pollen grains adhered to the stigma of the flowers (Table 2).

Pollen germination, on the other hand, was similar in percentage in all treatments in both experimental years, with values in all treatments around 30% one day after pollination in 2022 and 20% in season 2023. These levels of pollen germination decreased thereafter, especially 4 days after pollination, reducing the average value (Table 2). This abrupt decline in older flowers was not parallel to an increase in pollen adhesion, suggesting that the start of stigma senescence made it more difficult for us to ascertain pollen tube emission on the stigma surface.

In contrast, a big difference in self-pollinated flowers with respect to successful cross-pollination treatments was observed in the growth of the pollen tubes after penetrating the first cell layers of the stigma. However, some misunderstanding may appear when this process is labeled as a failure in pollen germination in the literature [32]. Pollen germination on the surface of the stigma is normally evaluated before squashing the pistil, as we did here, while the growth of pollen tubes inside the stigma and in the transmitting tissue of the style is measured on squashed flowers.

The observed inhibition of pollen tube growth led to significant reductions in fertilization levels (and therefore in fruit set) (Table 1 and Table 3) under self-pollination with respect to open-pollination and cross-pollination with Arbosana pollen in both years. Seed-paternity analyses actually confirmed that seed production under self-pollination treatment was in fact lower than the levels of fruit set because most of the seeds in this treatment were not a product of self-fertilization but rather the result of contamination with foreign pollen. Seed-paternity analyses showed also the failure of Arbequina to significantly father a good proportion of seeds, either under open-pollination or, more importantly, under the cross-pollination treatment in which we applied the pollen of Arbequina by hand (Table 4 and Table 5). A similar lack of success was also observed when we applied pollen of the supposedly unrelated cultivar Koroneiki (Table 5).

The high level of pollen contamination observed in these two cross-pollination treatments is possible because the procedure for cross-pollination involved repeatedly re-opening the isolation bags to perform cross-pollination, allowing the entrance of some foreign airborne pollen. This pollen contamination was undoubtedly proved by seed paternity tests. Pollen contamination with foreign airborne pollen has been frequently reported in wind-pollinated species, among them, olive [33,34,35,36]. In this regard, McGoey et al. [37] explain that for wind-pollinated plants, the challenge is either alter substantially the temperature and moisture inside the pollination bag, selecting airtight bags, or use very expensive material, not affordable for most field experiments. They check and recommend the use of individual growth chambers for controlling pollen flow in *Artemisia artemisiifolia*, but this procedure is not suitable for large olive trees. As a corollary, we emphasize the importance of preventing as much as possible pollen contamination by modifying pollination protocols as Saumitou-Laprade et al. [38] and Mariotti et al. [25] proposed and did using double bags and injecting the desired pollen with a needle in the second bag.

### 3.2. Self-Incompatibility System in Olive: GSI versus SSI

**Figure 2:**
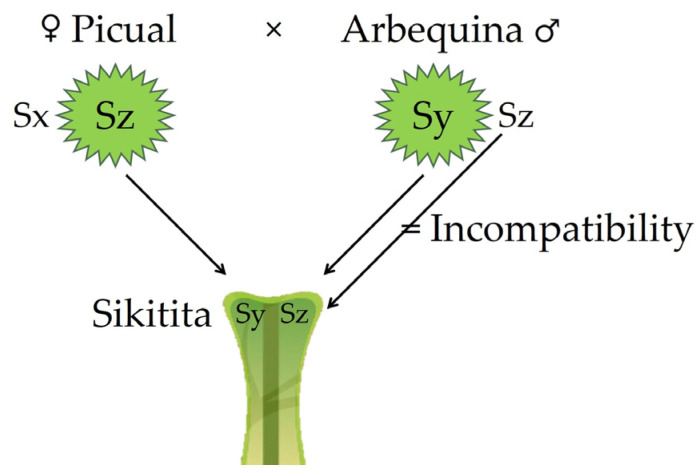
Singular and hypothetical S-genotypes for Sikitita and its parents (Picual as pollen recipient × Arbequina as pollen donor) that could only explain the backcross failure between Sikitita and its father Arbequina in a gametophytic self-incompatibility system.

If the SI response of Sikitita was expected, more surprising was the lack of affinity (inter-incompatibility) with its male parent Arbequina. The confirmed failure to backcross Sikitita with its male parent Arbequina demonstrated by pollination tests, by fruit set (in 2023), and by seed-paternity analyses strongly suggests that the SI system in olive is not of the type gametophytic (GSI). This is because even if half of the pollen grains of Arbequina might share the hypothetical S-allele with the Sikitita genotype, the remaining half population of pollen grains would grow without impediment in the pistil of Sikitita, achieving levels of fertilization and fruit set similar to those of other cross-pollination treatments. Nonetheless, the truth is that Arbequina pollen failed to successfully fertilize a large number of Sikitita flowers and to produce many seeds (although some were produced), even in the cross-pollination treatment in which Arbequina pollen was applied by hand. Only under very singular conditions could GSI explain the failure in backcrossing Sikitita and Arbequina. This could still be possible if both the male parent (Arbequina) and sibling (Sikitita) share both S-alleles, and that is only possible if the Sikitita father (Arbequina) and mother (Picual) share in turn one of the S-alleles (Figure 2). In stone fruit crops expressing GSI, the number of S-alleles is often between 20 and 40. If that were the case in *Olea europaea*, the chances for Sikitita and Arbequina having the same S-alleles would be certainly low. These unlikely circumstances (and previous results of different authors) make us to think that SI in olive is not GSI, but that olive expresses an SSI system.

### 3.3. Sporophytic Self-Incompatibility Systems in Olive: DSI versus PASI

Alternatively to GSI, different SSI systems have been proposed by Breton and Bervillé [16] and by Saumitou-Laprade et al. [18]. The main difference between these two models is that Breton and Bervillé [16] proposed a poliallelic SSI (PASI) with multiple crosses likely successful, while Saumitou-Laprade et al. [18] reduced the number of S-alleles to two in their diallelic SSI (DASI), the alleles S1 and S2, with a dominance of S2 over S1. In this last model, the authors grouped many olive genotypes in two groups: G1 with alleles S1S2, and G2, with genotype S1S1, compatible between them but incompatible with cultivars of the same group. Breton and Bervillé [16] proposed a dominance relationship expression of S-alleles in the male (pollen grains of the donor plant), but not in the female (pistil of the pollen receiving plant). Thus, a pair-wise combination of cultivars is postulated as inter-compatible in one direction but can be inter-incompatible in the other direction. Dominance relationships help these authors to explain certain levels of self-fertilization observed in many olive cultivars [39].

More recently, Breton et al. [40], trying to conciliate experimental work and some successful crosses within the two SI groups established by Saumitou-Laprade et al. [18], have proposed a dual SSI. These authors now acknowledge that the first loci would operate in the stigma as a DSI, inferring only two compatibility groups (G1 and G2), but a second loci would operate in the ovary, where pollen tube fates are controlled by a poly-allelic SI where up to 20 compatibility groups were suggested [40]. However, so far, no late rejection of pollen tubes has been reported in the ovary of *Olea europaea*. Therefore, it seems that DSI operating in the stigma of olive flowers has reached some consensus. Strong support of Saumitou-Laprade et al. [18] theory is that homomorphic DSI has been found to operate not only in other genera of the family Oleaceae like *Phillyrea*, *Fraxinus*, and *Ligustrum* [19,20,21], but also in the close relatives *Olea ferruginea* [41] and *Olea europaea* subsp. *laperrinei* [26]. Recent findings by Castric et al. [42] seem to confirm that in olive the SI system is a homomorphic DSI common to other Oleaceae, as they have located the self-incompatibility locus in a genome region largely conserved in the family Oleaceae and related to the gibberellin pathway.

However, some refinement seems necessary for the proposed DSI. For example, the authors have not provided a convincing reason to explain why there is not a third group of olive cultivars with genotype S2S2. Especially when, according to Mariotti et al. [25], S-alleles are inherited as Mendelian characters and, therefore, self-fertilization of cultivars of Group 1 (genotype S1S2) will produce 25% of the progeny with the genotype S2S2. Alagna et al. [32] correctly address this argument when exploring factors explaining pseudo-compatibility and explain that the appearance of a third homozygous group would lead to an imbalance of the olive plant population in favor of one group. We propose that a third group might exist, although the plant response may be dual: either pollen acceptance or rejection. The authors proposing DSI also concluded that cross-fertilization between cultivars of the same group would never happen either. However, in our experiments, self-fertilization, although reduced, occurred and cross-fertilization between cultivars belonging to the same incompatibility G1 (Sikitita × Arbequina, for instance) also happened often and both might produce 25% of the progeny with the genotype S2S2.

A second objection to DSI is that we expect around 50% of inter-incompatible crosses, and the abundant field experimentation carried out about SI in olive has documented far fewer cases of inter-incompatibility between cultivars. This question has been noted by different researchers [16,25]. Nonetheless, in previous studies, some olive cultivars were often labeled as “bad pollinator (sic)” in inter-specific crosses [28] and those may correspond to inter-incompatible crosses. In fact, pioneering and extensive work carried out by Riera [43] in 1950 did report many cases of supposedly inter-incompatible crosses in Spanish olive cultivars, although he did not provide any data nor describe the inter-incompatible reaction in any cross. On the other hand, pollination tests have shown more frequent cases of inter-incompatibility [14,25,44], than field studies. We recently reported that Arbosana is compatible with all three low-vigor cultivars tested as pollinizers of it [45], while in this experimentation, we found Sikitita to be inter-incompatible with Arbequina and Koroneiki and compatible with Arbosana, and likely Tosca. Since pollen contamination in olive flowers with foreign airborne pollen is much more frequent than initially thought, we do not discard that around 50% of crosses might indeed be inter-incompatible as Saumitou-Laprade et al. [18] propose.

Finally, heteromorphic SSI seems not to operate in olive despite differences observed in the size and shape of the stigma in some cultivars, and especially among different cultivars [7]. Saumitou-Laprade et al. [18] suggest that this topic merits some investigations. In this regard, very recently Raimondeau et al. [46] have found heteromorphic SSI in the family Oleaceae and reported that the same SI determinants operating in DSI control distyly in *Jasminum*. The role of exceptional male olive trees (female sterile), reputed as very good pollinizers but at risk of disappearance [43], has not been considered by any author to explain the evolution of the olive reproductive system as it was in *Phillyrea angustifolia* [30].

### 3.4. Incompatibility Group and S-Alleles for Sikitita

Anyway, the backcross failure between Sikitita and Arbequina provided some information about the possible incompatibility genotype and group to which Sikitita must be ascribed. In the DSI scheme earlier proposed by Saumitou-Laprade et al. [18], Mariotti et al. [25] assigned Arbequina to group G1 (genotype S1S2) and Picual to group G2 (genotype S1S1), as their successful cross may confirm. Then, two options emerge for Sikitita: if Sikitita were G1 (S1S2), a failure in the backcross between Sikitita and Arbequina is expected. If, on the contrary, Sikitita were G2 (S1S1), then we might expect full success for the backcross between Sikitita and Arbequina because of the dominance of S2 over S1. Although some levels of cross-fertilization occurred with Arbequina pollen, in our opinion, Sikitita should be assigned to G1, given the poor pollen tube growth found for Arbequina pollen grains on Sikitita pistils (Table 3), the significant reduction in fruit set in this treatment (Table 1), and the low number of seed products from this backcross (Table 4 and Table 5). The successful cross between Sikitita (as pollen donor) and Picual (as pollen recipient) reported by Klepo et al. [47] confirm that Sikitita belongs to group G1 and Picual to group G2.

On the other hand, Mariotti et al. [25] assigned Koroneiki to group G2, a circumstance that should make the crossing of Sikitita × Koroneiki successful. On the contrary, our results showed similar failure for Koroneiki pollen to fertilize a large number of Sikitita flowers. That is, if the preliminary assignment of Sikitita to G1 is correct, then the crosses with Koroneiki should be fully successful, but the truth is that Koroneiki pollen failed to increase fertilization with respect to self-pollination (Table 3). Furthermore, pollen tube growth syndrome in Sikitita × Koroneiki treatment clearly resembles that of inter-incompatible crosses (Table 3). Additionally, we recently probed in parallel studies that Koroneiki is a very good pollinizer for Arbosana (G2) (as Arbequina (G1) is) [45]. Therefore, Sikitita and Koroneiki seem to belong to the same incompatibility group, and the latter should be preliminary ascribed to G1. Breton and Bervillé [16] assigned the genotype R1R3 to Arbequina, but no information about Picual is given. Therefore, we cannot explore the possible genotype of their sibling Sikitita according to the model these last authors initially proposed.

### 3.5. Implications of Self-Fertilization Occurrence on SSI Models

DSI model proposed by Saumitou-Laprade et al. [18] rejects, in theory, the possibility of self-fertilization and fertilization among cultivars of the same incompatibility group. However, the truth is that self-fertilization occurs, as the previous authors themselves acknowledge, although they restricted occasional self-fertilization to G2. In this regard, our results, and many others before, probe that in some cases, pollen tubes escape from SI rejection in the stigma of olive flowers, and although delayed, pollen tubes are able to cross the style and reach the ovule without any additional obstacle.

In the opinion of Breton and Bervillé [16], dominance relationships, as that observed in guayule, might explain different levels of self-pollination observed in several olive cultivars. Reduced, but proved, occurrence of self-fertilization in olive can be explained by different physiological mechanisms widely described by de Nettancourt [31]. The mentor effect is likely the better-known mechanism in other species thanks to their use in breeding programs to facilitate self-fertilization. In the mentor effect, cross-pollen grains (irradiated or not) act as pioneers in a forward line, opening the route toward the ovules to self-pollen grains, allowing thus certain levels of self-fertilization. This mechanism cannot explain our results since cross-pollen was not applied in bagged self-pollinated flowers, where some self-fertilization took place. A different mechanism often cited to procure self-fertilization is the massive deposition of self-pollen to saturate the enzymes implicated in the SI reaction. This massive self-pollen deposition did not happen in our experiments either, as the pollen adhesion data indicate (Table 2). A third explanation for what is sometimes called pseudo-compatibility is pistil aging, a mechanism by which old senescent flowers are more permissive and tolerate some self-pollen growth in the pistil (and self-fertilization too) in the absence of cross-pollen, always preferred when present. This last mechanism allows some reproductive success in isolated plants and matches observations of late pollen tube growth in self-pollinated flowers of olive.

In this regard, in several experiments carried out in olive, it has been common to observe not only low levels of fertilization but also a delayed growth of pollen tubes in self-pollination [4,6], suggesting that early arrest to self-pollen is not maintained in time. This delayed pollen tube growth explains the reduced and late levels of self-fertilization often found in olive, allowing at the same time some preference for cross-pollen. Although this reproductive assurance strategy makes biological and evolutionary sense [48], self-pollination tests carried out on increasingly older virgin flowers of Manzanillo (syn. Manzanilla de Sevilla) failed to cause increasing levels of self-fertilization in older flowers of this strongly self-incompatible cultivar and/or a minor self-pollen rejection (Cuevas, unpublished results). Alagna et al. [32] also deny that flower age may explain pseudo-compatibility occurrence. On the contrary, the less extreme SI response in irrigated olive trees and in “off” years, in which ovule longevity and stigma receptivity are prolonged [49,50], is compatible with delayed self-pollen tube growth and some levels of self-fertilization. However, much more research is needed to better document self-pollen growth in old olive flowers.

Finally, Breton et al. [39] suggest a genetic basis for pseudo-fertility in some olive cultivars, proposing a complicated dominance relationship of some alleles over others (R6 > R2 > R1 = R3 = R5 > R4), among the six S-alleles they deduce are present in olive. In their theory, different levels of self-compatibility (or pseudo-compatibility) are possible, with genotypes carrying R1 alleles being more self-incompatible than those carrying R5, while genotypes with allele R3 show an intermediate response.

### 3.6. Effects of Different SI Models on Pollination Designs

SI systems have a strong influence on pollination designs and pollinizer choice. In GSI, the crosses in both directions are either incompatible or compatible, with no differences depending on the direction. This is the reason why the pollination design in the orchards of pome and stone fruit crops of the family Rosaceae often includes only two cultivars that successfully cross-pollinate each other. The high number of S-alleles in these crops (more than 20) allows plenty of opportunities to choose pollinizers for the main cultivar, as the mating availability is high.

In contrast, in the DSI model proposed by Saumitou-Laprade et al. [38], the choice of pollinizers is much more restricted (roughly to half the population) since the authors propose the existence of two groups equally represented, within which the cultivars are incompatible between them. The existence of a hypothetical third group, not yet proven, with genotype S2S2, would increase the possibilities of successful crosses for cultivars belonging to the group G2 (S1S1). Crosses between G1 cultivars (S1S2) would also be incompatible with this third hypothetical new group (S2S2) due to the proposed dominance of the allele S2. On the other hand, Saumitou-Laprade et al. [18] and Mariotti et al. [25] did not find any asymmetry in the incompatibility reaction depending on the cultivars acting as pollen donors or pollen recipients. Wu et al. [14] and Cuevas et al. [27] found reciprocal effects when performing diallelic crosses to select suitable pollinizers for the main Spanish, Italian, and Greek cultivars. The recent release of a new promising olive cultivar named Sultana [51], a product of the cross between Arbosana (female pollen-recipient parent) and Sikitita (male pollen-donor parent), confirms that the compatibility between them is bidirectional. We recently found also a common response and good compatibility between Sikitita and Arbosana [45], and that seems to be the norm.

On the contrary, in the model proposed by Breton and Bervillé [16], a pair combination of varieties may be inter-compatible in one direction and inter-incompatible in the other direction. In this regard, Breton et al. [52] identified, based on the literature, a high number of asymmetric crosses, compatible in one direction but inter-incompatible in the other. If the results depend on the direction of the cross (male-sterile olive cultivars apart), then one pollinizer could not be enough for appropriate olive pollination designs. The reason is that, although the selected pollinizer might fertilize the flowers of the main cultivar, it is not certain, in their model, that the pollen of the main cultivar will act the same and successfully fertilize the flowers of the pollinizer. Introducing three cultivars in the pollination design makes it more difficult and likely less profitable in olive crops, especially in high-density orchards. Nonetheless, most of the results obtained in olive suggest that a pollination design with two inter-compatible cultivars may be enough.

## 4. Materials and Methods

### 4.1. Experimental Site and Pollination Treatments

**Figure 3:**
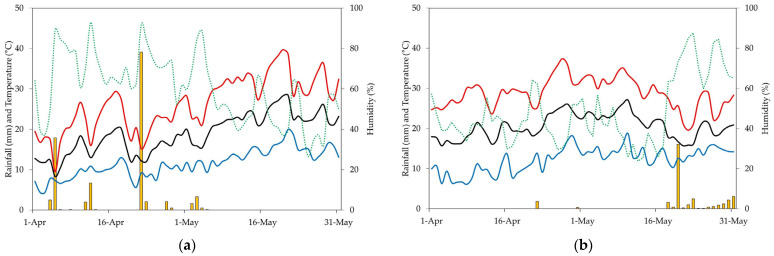
Temperature (maximum, minimum and average, in red, blue and black lines, respectively) average humidity (green line), and rainfall (yellow bars) during the experimental seasons 2022 (**a**) and 2023 (**b**). Data retrieved from a weather station sited in the experimental area at the Campus of the University of Córdoba.

The experiments were carried out in two consecutive seasons (2022 and 2023) in a super-high density orchard located on the Rabanales Campus of the University of Córdoba (Córdoba, Spain; 37°56′05″ N, 4°43′00″ W, at 160 m altitude). According to Papadakis [53] classification, the area has a subtropical Mediterranean climate, with warm and dry summers and mild and wet winters. The average annual temperature is 18.4 °C (average maximum temperature = 25.5 °C, average minimum temperature = 11.4 °C). The accumulated annual rainfall is around 570 mm. Average relative humidity ranges between 40 and 80%. The hours of sunlight reach an average value of 2903 h per year. The weather during bloom season and fruit setting periods in 2022 and 2023 is shown in Figure 3.

As each olive cultivar consists of clones of the same genotype, we selected, as replications, four homogeneous Sikitita trees of the same size and all in their “on” year. On each replication, we applied four pollination treatments in the first season: self-, open-, and cross-pollination with the male parent genotype (Arbequina), and cross-pollination with the unrelated cultivar Arbosana. In the second year (season 2023), we added one more cross-pollination treatment using Koroneiki (another low-vigor cultivar also grown in the orchard), a genotype from Greece and likely unrelated to Sikitita. The pollination treatments were applied to eight (year 2023) or ten (year 2022) 1-year-old shoots, distributed all around the tree canopy at the observer’s height. Each tagged shoot bore 10 panicles; this uniform level of flowering was achieved by removing the panicles in excess when necessary to avoid the interference of different flowering loads with incompatibility reactions.

Self-pollination was achieved by bagging individually each shoot before bloom with tissue paper bags made by hand. This material, although fragile in the event of rain, is preferred because it scarcely modifies the environmental conditions inside the bag. Cross-pollination treatments were performed by applying fresh pollen grains from the corresponding genotypes collected from nearby trees with a camel paintbrush. Cross-pollination was performed on open flowers every other day and at least three times during the bloom period. Open-pollinated flowers were left unbagged and exposed to pollen arrival from different sources during the whole bloom period. The flowers were not emasculated, as in nature, so flowers may receive self-pollen in addition to the corresponding pollination treatment.

### 4.2. Initial and Final Fruit Set Measurements

In these shoots, we measured both the initial and final fruit sets. The initial fruit set was measured between 2 and 3 weeks after bloom (16 and 20 days) depending on the year, counting the number of enlarged fruitlets per shoot. The final fruit set was measured 7 weeks after bloom, once the period of fruitlet competition (“June” drop) has ended and the fruit population is established in olive. Final fruit set is a good predictor of yield at harvest; later fruit drop may occur in response to accidents, pests, and diseases, but the losses are not related to pollination treatments. Initial and final fruit set was expressed as fruitlets per panicle, since the panicle is the unit of fructification in olive. Finally, we calculated the index of self-incompatibility (ISI) as proposed by Zapata and Arroyo [54], dividing the self-pollination final fruit set by cross-pollination (and open-pollination) values from the final fruit set.

### 4.3. Pollen–Pistil Interaction Measurement

In an additional set of flowers, but on different shoots of the experimental trees, the same pollination treatments were applied to analyze the following pollen–pistil interaction processes: pollen adhesion, pollen germination, pollen tube growth, and ovule fertilization. Sets of 20 flowers per treatment (occasionally fewer) were sampled 1, 2, 4, and 8 days after pollination. Pollination was always performed during anthesis. The day of anthesis was established by removing one day all open flowers of the sample and the closed flowers the next day; so, all flowers opened between these two consecutive days. On the sampling dates, the sets of flowers were collected in a vial and immediately fixed in the field in a FAE solution (formalin, acetic acid, and 70% ethanol at a volume ratio of 1:2:17). From the field, the samples were taken to the lab and kept in a fridge at 4 °C until observation under fluorescence microscopy. For the analyses, the flowers were softened during 8 h with a solution of NaOH 1N, rinsed overnight in running tap water, stained with aniline blue, and gently squashed for observation under fluorescence microscopy using a Labophot microscope (Nikon, Tokyo, Japan) following the procedure described by Cuevas et al. [3].

**Figure 4:**
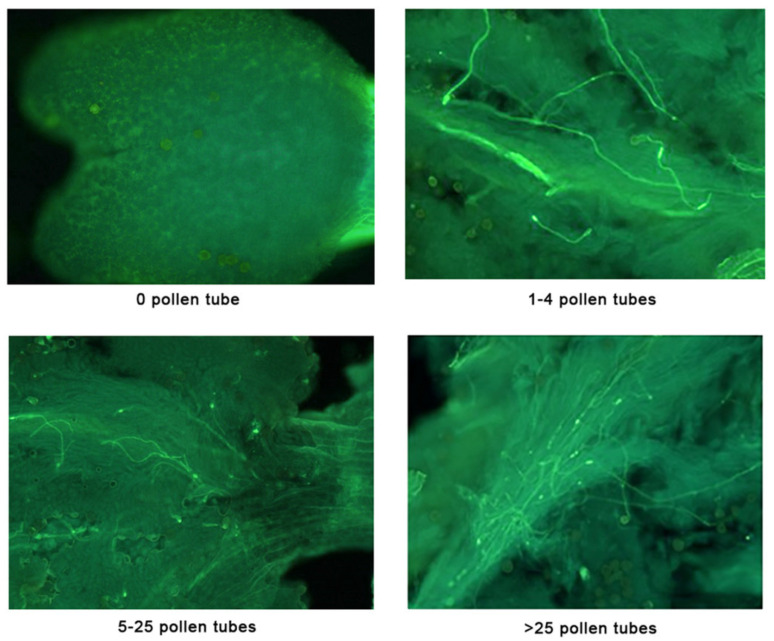
Scale from 0 to 3 to quantify pollen tube growth based on the number traversing the stigma and style. Published by Sánchez-Estrada and Cuevas [55]. With Permission of HortTechnology, a Journal of the American Society for Horticultural Science. Before squashing, the length of the stigma was in the range of 1–1.3 mm, and width of stigma between 0.7 and 0.9 mm.

Pollen adhesion and germination were studied 1, 2, and 4 days after pollination since the start of stigma senescence makes estimation of germination in older flowers (8 days after pollination) more difficult. Given the small size of the stigma in Sikitita flowers, all pollen grains on one side of the stigma were counted, and the total number was estimated by multiplying by two these records. Pollen grains were considered germinated when the grains emitted a pollen tube at least as long as the pollen grain diameter. Pollen adhesion and germination were measured on the surface of the stigma before squashing the flowers for the evaluation of pollen tube growth inside the pistil. The pollen tube growth within the stigma and style was characterized using a scale from 0 to 3, where 0 means no pollen tubes present, 1 means a reduced number (1 to 4) of pollen tubes growing in the upper part of the style, 2 means between 5 and 25 pollen tubes in the style, and 3 means more than 25 pollen tubes (Figure 4). After dissecting the four ovules from the flowers, fertilization was measured and expressed as the percentage of flowers with at least one (usually only one) ovule fertilized. This was ascertained when a pollen tube was present in the micropyle of the ovule.

### 4.4. Seed Paternity Determination

To determine the paternity of the seed embryos, genomic DNA was extracted from the embryos collected from the mother trees and from the leaves of the potential pollen donor cultivars. The NucleoSpin™ Plant II kit (Machery-Nagel, Dueren, Germany) was used for the extraction of genomic DNA according to the manufacturer’s instructions. Eight polymorphic SSR markers were used for the genotyping of the embryos and of the potential pollen donors: ssrOeUA-DCA-(3, 5, 15, 18) [56], GAPU-(71B, 101) [57], and EMO-(3, 90) [58]. The PCR reaction was carried out in a final volume of 12.5 µL with the composition and conditions described by Cuevas et al. [38]. The PCR products were diluted to 1 µL and analyzed on a SeqStudio™ Genetic Analyzer (Thermo Fisher Scientific, Marsiling, Singapore). Electropherograms were checked using GeneMapper version 5 software (Thermo Fisher Scientific). Five potential pollen donors (Tosca, Sikitita, Koroneiki, Arbequina, and Arbosana) equally represented in the orchard were genotyped to obtain the reference profiles. Afterwards, the same genotyping procedure was applied to all embryos obtained from open-pollination and for those from self-pollination or cross-pollination treatments in which the presumed father alleles were not assigned. Microsatellite information and paternity assignment were performed using CERVUS 3.0 software (http://www.fieldgenetics.com; accessed on 30 June 2024) [59]. Each CERVUS run consisted of an analysis of allele frequency followed by a simulation in which the number of candidate fathers was set at five (the cultivars present in the orchard), while the proportion of candidate fathers sampled was set to a confidence level of 80%. The proportion of typed loci was set to the percentage estimated by the allele frequency analysis. A minimum of four typed loci were required for the progeny to be analyzed for paternity, and 100,000 offspring were simulated. The genotyping error in the simulation and in the assignment of the most likely pollen donor was set at 1%. When no assigned fathers could be established for progeny among the cultivars present in the experimental orchard, it was presumed that the pollen belonged to olive cultivars from surrounding orchards.

### 4.5. Statistical Analyses

The effects of the different pollination treatments on the fruit set and the fertilization levels were analyzed by analyses of variance, with the means compared when needed using Tukey’s test (*p* < 0.05) using Statistix 8.0 (Analytical Software, Tallahassee, FL, USA).

## 5. Conclusions

Our results demonstrate that Sikitita olive is strongly self-incompatible. As proved in many olive cultivars before, the SI reaction is characterized by the rejection of self-pollen in the stigma soon after germination. Self-pollen rejection leads to reduced fertilization and fruit set. Nonetheless, that does not imply that self-fertilization was completely impossible, and, although seed-paternity analyses confirm the extreme self-incompatibility of Sikitita, it confirmed the occurrence of a reduced level of self-fertilization. On the contrary, cross-pollination with Arbosana pollen was highly successful with massive pollen tube growth, high levels of ovule fertilization, and good fruit set. Seed paternity analyses confirm that Arbosana pollen fathered a high proportion of embryos in the fruit produced in that cross-pollination treatment, but also under open pollination and, surprisingly, in other pollination treatments in which Arbosana pollen improperly managed to gain access to Sikitita flowers. Tosca also fathered a considerable number of Arbequina seeds, suggesting that Tosca is inter-compatible with Sikitita too. On the contrary, the backcrossing of Sikitita with its father Arbequina was a failure. This result informs of their inter-incompatibility and suggests that olive possesses an SSI system. Nonetheless, although very unlikely, GSI cannot be completely discarded based solely on our results.

Pollen–pistil interaction under self-pollination in Sikitita showed the SI reaction characterized by the blockage of self-pollen soon after penetration of the first cell layers of the stigma. Consequently, we endorse the stigma test used by Saumitou-Laprade et al. (2017) [18] as a rapid screening method to establish self- and inter-incompatibility responses. However, we propose using our 0–3 scale to better describe, measure, and compare pollen tube growth in the different crosses. In addition, we strongly suggest using fresh, not frozen, pollen, or alternatively, controlling pollen viability by reliable methods. More importantly, we advise against the use of in vitro pollination with detached flowers (in their case, just three flowers per cross) placed on agar in the lab, since olive expresses strong cavitation problems that precluded the use of detached flowers for pollination experiments [2]. On the contrary, we recommend that, as usual, pollination can be performed in vivo by bagging the inflorescences days before anthesis and opening the isolation bags just once to apply the desired pollen before collecting the flowers and fixing them. We also suggest measuring pollen tube growth 2 and 4 days after pollination and not just 16 h after, to avoid any interference with late stigma receptivity or a slight dichogamous protandry present in some olive cultivars.

Finally, we recommend extending the pollination experiments and performing backcrossing of new genotypes produced by different olive breeding programs, especially those designed for large, high-density orchards. We also strongly suggest that compatibility studies between olive cultivars must always include pollination tests, fruit set (or yield) data, and seed paternity determinations.

## Figures and Tables

**Table 1:** Sikitita fruit set in response to different pollination treatments. Seasons 2022 and 2023.

Pollination Treatments	Season 2022			Season 2023		
	Initial Fruit Set *	Final Fruit Set	ISI **	Initial Fruit Set	Final Fruit Set	ISI
Self-pollination	0.08 c ***	0.05 c		0.12 c	0.11 b	
Open-pollination	1.18 a	0.68 a	0.07	0.98 a	0.61 a	0.18
×Arbequina	0.60 b	0.45 b	0.11	0.27 bc	0.25 b	0.44
×Arbosana	0.95 a	0.63 ab	0.08	0.85 a	0.50 a	0.22
×Koroneiki	–	–	–	0.58 ab	0.50 a	0.22
*p*	<0.0001	<0.0001		0.0012	0.0002	

* Fruit set = fruit per panicle 2 and 7 weeks after bloom, measured in 8–10 shoots per replication (tree) each one bearing an equal flowering load of 10 panicles. ** ISI (index of self-incompatibility) = Final fruit set under self-pollination/final fruit set under open- or cross-pollination. *** In the columns, means followed by different letters are significantly different according to ANOVA at *p* ≤ 0.05. Separations of means by Tukey test.

**Table 2:** Pollen adhesion and germination on Sikitita stigmas under different pollination treatments in seasons 2022 and 2023. Average ± standard error of the values measured 1, 2, and 4 days after pollination.

Pollination Treatments	Season 2022		Season 2023	
	Pollen Adhesion *	Pollen Germination **	Pollen Adhesion *	Pollen Germination **
Self-pollination	442 ± 42	78 ± 12	361 ± 46	44 ± 6
Open-pollination	233 ± 26	40 ± 4	343 ± 52	53 ± 8
×Arbequina	395 ± 37	56 ± 8	274 ± 31	30 ± 4
×Arbosana	302 ± 31	57 ± 8	576 ± 64	60 ± 5 ***
×Koroneiki	–	–	358 ± 46	34 ± 6

* Pollen grains adhered per stigma. ** Pollen grains germinated on the surface of the stigma. *** Samples from 2 days after pollination are missing in season 2023. Average of values at 1 and 4 days after pollination in this case.

**Table 3:** Pollen tube growth and fertilization levels in Sikitita flowers in response to different pollination treatments. Seasons 2022 and 2023. Average ± standard error of pollen tube growth measured 2, 4, and 8 days after pollination. Fertilization values compared by chi square tests.

Pollination Treatments	Season 2022		Season 2023	
	Pollen Tube Growth *	Fertilization **	Pollen Tube Growth *	Fertilization **
Self-pollination	0.07 ± 0.04	0.0 c ***	0.15 ± 0.06	12.7 c
Open-pollination	1.12 ± 0.16	16.9 ab	1.50 ± 0.19	31.4 b
×Arbequina	0.42 ± 0.16	10.0 b	0.13 ± 0.06	16.4 bc
×Arbosana	1.76 ± 0.19	28.8 a	2.20 ± 0.15	60.0 a
×Koroneiki	–	–	0.25 ± 0.07	15.4 bc

* Pollen tube growth measured in a scale from 0 (absence of pollen tubes growing in the upper part of the style) to 3 (more than 25 pollen tube present in the upper part of the style) (see Figure 4). ** Percentage of flowers with a pollen tube in the micropyle of at least one of the four ovules. *** In the columns of fertilization, means followed by different letters are significantly different at *p* ≤ 0.05.

**Table 4:** Number of embryos in Sikitita mother trees assigned to each pollen genotype (columns) in the different pollination treatments during the 2022 season. LOD score threshold = 0.40. In bold, the most successful fathers.

Pollination Treatment	Sikitita *	Arbequina	Arbosana	Koroneiki	Tosca	Unknown Cultivar **	Below LOD Value
Self-pollination	**6**	0	**4**	1	0	0	2
Open-pollination	0	6	**26**	1	4	0	32
×Arbequina	0	9	**37**	0	0	0	9
×Arbosana	0	0	**46**	0	0	1	0

* ×Sikitita represents self-fertilization. ** SSR markers profile does not correspond to any of the five cultivars present in the experimental orchard. Fertilization by a different genotype of pollen applied denotes some degree of pollen contamination in self- and cross-pollination treatments.

**Table 5:** Number of embryos in Sikitita mother trees assigned to each pollen genotype (columns) in the different pollination treatments during the 2023 season. LOD score threshold = 0.89. In bold, the most successful fathers.

Pollination Treatment	Sikitita *	Arbequina	Arbosana	Koroneiki	Tosca	Below LOD Value
Self-pollination	1	0	**28**	1	8	5
Open-pollination	0	8	**37**	9	7	16
×Arbequina	0	2	**5**	1	**6**	6
×Koroneiki	0	1	**8**	1	**6**	7

* ×Sikitita represents self-fertilization. Fertilization by a different genotype of the pollen applied denotes some degree of pollen contamination in self- and cross-pollination treatments.

## Data Availability

The raw data supporting the conclusions of this article will be made available by the authors on reasonable request.

## Supplementary Materials

The following supporting information can be downloaded at: https://www.mdpi.com/article/10.3390/plants13202872/s1, Table S1: List of Sikitita seeds’ embryos with LOD values below threshold (0.40), their genotype profiles, pollination treatment and replication, and most likely a male parent during 2022 season; Table S2: List of Sikitita seeds’ embryos with LOD values below threshold (0.89), their genotype profiles, pollination treatment and replication, and most likely a male parent during 2023 season.
