# Supplementary material for: Backcrossing Failure between Sikitita Olive and Its Male Parent Arbequina: Implications for the Self-Incompatibility System and Pollination Designs of Olive Orchards
Source: Plants (Basel). 2024 Oct 14;13(20):2872. doi: 10.3390/plants13202872 (PMC11510835; doi:10.3390/plants13202872)
Supplement: Supplementary file 1 [file plants-13-02872-s001.zip › Supplementary-TableS1-Sikitita2022.pdf]

Table S1. List of Sikitita seeds' embryos with LOD values below threshold (0.40), their genotype profiles, pollination treatment and replication, and more likely male parent. Season 2022.

No	DCA5	DCA5	EMO3	EMO3	DCA3	DCA3	GAPU101	GAPU101	GAPU71B	GAPU71B	EMO90	EMO90	DCA18	DCA18	DCA15	DCA15	TREATMENT	LIKELY FATHER	LOD SCORE
1	204	204	215	215	236	246	199	218	120	130	184	186	169	177	243	263	Open-pollination R1	Arbosana	below 0.40
3	194	204	209	211	236	236	186	194	124	130	186	186	169	175	254	254	Open-pollination R1	Koroneiki	below 0.40
6	204	204	215	215	236	246	186	201	124	144	186	186	169	179	243	254	Open-pollination R1	Tosca	below 0.40
9	196	204	211	211	236	240	186	186	120	124	186	186	169	177	243	254	Open-pollination R1	Arbosana	below 0.40
10	204	204	211	211	240	240	186	186	120	124	186	186	169	177	254	266	Open-pollination R1	Arbosana	below 0.40
14	204	204	215	215	228	240	186	186	124	130	186	186	169	181	254	263	Open-pollination R1	Arbequina	below 0.40
15	204	204	211	219	228	236	192	192	120	130	186	196	169	187	254	263	Open-pollination R2	Arbosana	below 0.40
18	204	204	215	219	240	246	201	218	120	124	186	186	169	177	243	254	Open-pollination R2	Arbosana	below 0.40
19	204	204	215	219	240	246	201	218	120	124	186	186	169	177	243	254	Open-pollination R2	Arbosana	below 0.40
20	204	204	211	215	240	246	186	237	124	144	186	186	177	177	243	254	Open-pollination R2	Tosca	below 0.40
21	204	204	215	215	236	240	186	237	120	130	186	186	169	177	243	263	Open-pollination R2	Arbosana	below 0.40
22	204	204	211	215	240	242	186	237	120	130	186	186	169	173	254	263	Open-pollination R2	Arbosana	below 0.40
23	204	204	211	211	236	240	186	237	120	124	186	186	171	177	254	254	Open-pollination R2	Tosca	below 0.40
25	204	204	211	211	236	246	192	218	120	130	186	186	177	181	263	263	Open-pollination R2	Arbosana	below 0.40
27	196	204	215	215	236	236	186	186	126	130	186	186	173	177	243	263	Open-pollination R2	Koroneiki	below 0.40
28	204	204	211	211	236	240	186	186	124	144	186	186	169	169	243	263	Open-pollination R2	Arbosana	below 0.40
29	204	204	211	213	236	240	237	237	130	144	186	186	171	177	254	254	Open-pollination R2	Tosca	below 0.40
33	204	204	215	219	236	250	186	201	120	124	186	186	169	177	263	263	Open-pollination R3	Arbequina	below 0.40
36	204	204	211	219	240	250	186	194	124	126	186	196	169	188	263	263	Open-pollination R3	Arbequina	below 0.40
37	204	204	211	213	236	246	194	218	124	130	186	186	177	181	254	263	Open-pollination R3	Koroneiki	below 0.40
40	204	204	211	211	236	250	186	186	124	124	186	186	177	181	243	263	Open-pollination R3	Tosca	below 0.40
42	204	204	211	211	240	240	186	186	120	130	186	186	169	177	243	254	Open-pollination R3	Arbosana	below 0.40
48	204	204	211	219	236	240	186	194	120	124	186	186	177	177	254	263	Open-pollination R3	Tosca	below 0.40
49	204	204	211	215	236	242	218	218	120	130	186	186	169	181	243	263	Open-pollination R3	Arbosana	below 0.40
50	204	208	211	211	226	240	186	218	124	130	186	186	169	169	243	254	Open-pollination R3	Tosca	below 0.40
53	204	204	211	215	236	240	186	192	120	124	186	188	169	169	243	254	Open-pollination R4	Arbosana	below 0.40
54	204	204	211	215	236	236	186	192	120	130	186	186	169	177	243	263	Open-pollination R4	Arbosana	below 0.40
56	204	204	211	215	236	240	199	218	124	124	186	186	171	177	254	263	Open-pollination R4	Tosca	below 0.40
60	204	204	211	215	236	240	192	218	120	130	184	186	169	177	254	266	Open-pollination R4	Arbosana	below 0.40
62	204	204	211	215	240	246	192	218	120	130	186	186	169	177	243	254	Open-pollination R4	Arbosana	below 0.40
66	204	204	211	219	240	246	186	186	120	130	186	186	169	171	254	263	Open-pollination R4	Arbosana	below 0.40
No	DCA5	DCA5	EMO3	EMO3	DCA3	DCA3	GAPU101	GAPU101	GAPU71B	GAPU71B	EMO90	EMO90	DCA18	DCA18	DCA15	DCA15	TREATMENT	LIKELY FATHER	LOD SCORE

No	DCA5	DCA5	EMO3	EMO3	DCA3	DCA3	GAPU101	GAPU101	GAPU71B	GAPU71B	EMO90	EMO90	DCA18	DCA18	DCA15	DCA15	TREATMENT	LIKELY FATHER	LOD SCORE
67	204	204	211	211	236	240	192	218	120	124	184	186	169	177	243	263	Open-pollination R4	Arbosana	below 0.40
78	204	204	211	219	236	248	186	192	130	130	186	186	173	177	243	254	Self-pollination R3	Tosca	below 0.40
82	204	204	215	215	236	250	192	199	124	124	186	188	177	177	254	254	Self-pollination R4	Tosca	below 0.40
86	204	204	211	215	228	240	186	186	120	124	186	186	169	177	243	263	x Arbequina R1	Arbosana	below 0.40
94	204	204	211	211	236	240	186	186	120	124	186	186	169	181	243	254	x Arbequina R2	Arbosana	below 0.40
96	204	204	211	215	228	240	186	186	120	130	186	186	169	177	243	263	x Arbequina R2	Arbosana	below 0.40
100	204	204	211	215	236	240	186	218	120	124	186	186	169	181	254	266	x Arbequina R2	Arbosana	below 0.40
101	204	204	215	215	240	240	186	186	120	130	186	186	169	177	254	266	x Arbequina R2	Arbosana	below 0.40
102	204	204	211	215	236	240	192	192	120	130	184	186	169	177	243	254	x Arbequina R2	Arbosana	below 0.40
105	204	204	211	215	228	236	192	192	124	130	184	186	169	169	243	263	x Arbequina R2	Arbosana	below 0.40
114	204	204	211	215	240	240	186	192	130	144	184	186	169	181	254	254	x Arbequina R3	Arbosana	below 0.40
123	204	204	211	213	240	250	186	192	124	144	186	186	169	181	263	263	x Arbequina R4	Arbosana	below 0.40
**No**	**DCA5**	**DCA5**	**EMO3**	**EMO3**	**DCA3**	**DCA3**	**GAPU101**	**GAPU101**	**GAPU71B**	**GAPU71B**	**EMO90**	**EMO90**	**DCA18**	**DCA18**	**DCA15**	**DCA15**	**TREATMENT**	**LIKELY FATHER**	**LOD SCORE**
