# Supplementary material for: Backcrossing Failure between Sikitita Olive and Its Male Parent Arbequina: Implications for the Self-Incompatibility System and Pollination Designs of Olive Orchards
Source: Plants (Basel). 2024 Oct 14;13(20):2872. doi: 10.3390/plants13202872 (PMC11510835; doi:10.3390/plants13202872)
Supplement: Supplementary file 1 [file plants-13-02872-s001.zip › Supplementary-TableS2-Sikitita2023.pdf]

Table S2. List of Sikitita seeds' embryos with LOD values below threshold (0.89), their genotype profiles, pollination treatment and replication, and more likely male parent. Season 2023.

No	DCA5	DCA5	EMO3	EMO3	DCA3	DCA3	GAPU101	GAPU101	GAPU71B	GAPU71B	EMO90	EMO90	DCA18	DCA18	DCA15	DCA15	TREATMENT	LIKELY FATHER	LOD SCORE
6	204	204	213	215	240	248	186	205	124	126	186	186	177	188	243	254	Open-pollination R1	Tosca	below 0.89
9	204	204	215	215	236	250	186	199	130	144	186	192	177	177	263	263	Open-pollination R1	Arbequina	below 0.89
14	198	204	211	215	236	236	186	218	124	126	186	186	169	188	254	266	Open-pollination R1	Arbosana	below 0.89
17	204	208	211	215	236	240	186	199	124	144	186	186	175	177	243	254	Open-pollination R1	Tosca	below 0.89
19	204	204	213	215	236	242	194	218	124	130	186	186	169	173	254	254	Open-pollination R2	Koroneiki	below 0.89
25	204	204	211	213	236	248	186	205	124	124	186	188	177	189	243	254	Open-pollination R2	Tosca	below 0.89
26	196	204	211	215	240	250	186	199	124	130	186	188	171	177	243	263	Open-pollination R2	Tosca	below 0.89
31	200	204	215	215	236	246	186	192	124	130	186	186	173	177	254	263	Open-pollination R2	Arbequina	below 0.89
33	204	210	209	215	236	242	186	194	124	126	186	186	171	177	263	263	Open-pollination R2	Koroneiki	below 0.89
35	204	204	211	211	236	246	186	194	120	124	186	186	169	177	243	254	Open-pollination R2	Arbosana	below 0.89
44	196	204	215	215	236	240	207	218	126	130	186	186	169	188	263	263	Open-pollination R3	Arbequina	below 0.89
54	204	204	213	215	234	240	186	218	120	130	186	186	177	181	254	263	Open-pollination R3	Tosca	below 0.89
58	204	212	211	211	236	236	186	218	124	126	186	186	173	177	243	263	Open-pollination R4	Tosca	below 0.89
59	198	204	211	215	234	240	192	218	124	147	184	186	163	177	254	266	Open-pollination R4	Arbosana	below 0.89
67	204	204	215	215	236	250	218	220	124	144	186	186	169	181	243	254	Open-pollination R4	Arbosana	below 0.89
74	204	204	211	215	240	250	186	194	130	144	186	186	177	181	254	263	Open-pollination R4	Arbosana	below 0.89
78	204	204	211	215	236	242	186	218	124	144	186	188	173	177	254	263	x Arbequina R1	Tosca	below 0.89
81	204	204	211	215	226	236	186	186	130	144	186	188	177	177	243	254	x Arbequina R1	Tosca	below 0.89
83	204	204	211	215	236	242	186	199	124	144	186	188	169	173	254	263	x Arbequina R2	Tosca	below 0.89
87	204	204	211	215	236	246	194	218	130	130	186	186	169	177	263	263	x Arbequina R2	Koroneiki	below 0.89
94	204	204	213	215	236	236	194	218	124	130	186	186	169	173	243	263	x Arbequina R4	Koroneiki	below 0.89
97	204	204	211	215	240	250	186	199	124	144	186	188	169	173	263	263	x Arbequina R4	Tosca	below 0.89
108	204	204	211	213	240	246	186	199	124	144	186	188	171	177	243	263	Self-pollination R1	Tosca	below 0.89
110	200	204	215	219	236	246	194	218	124	126	186	186	169	171	243	254	Self-pollination R2	Arbequina	below 0.89
113	204	204	211	215	240	242	199	218	130	144	186	188	169	171	263	263	Self-pollination R2	Tosca	below 0.89
129	204	204	211	215	240	248	194	218	124	130	186	186	169	188	243	254	Self-pollination R3	Tosca	below 0.89
137	204	212	211	215	228	240	218	218	124	126	186	186	169	177	243	263	Self-pollination R4	Arbequina	below 0.89
144	204	204	211	215	236	238	186	194	124	130	186	186	169	171	243	263	x Koroneiki R1	Tosca	below 0.89
147	204	204	211	215	238	246	194	218	124	124	186	186	169	175	254	263	x Koroneiki R1	Koroneiki	below 0.89
151	198	204	213	215	228	236	186	186	124	144	186	186	163	169	254	263	x Koroneiki R2	Arbequina	below 0.89
157	204	204	211	219	236	240	194	218	124	130	186	186	169	177	263	266	x Koroneiki R3	Arbosana	below 0.89
**No**	**DCA5**	**DCA5**	**EMO3**	**EMO3**	**DCA3**	**DCA3**	**GAPU101**	**GAPU101**	**GAPU71B**	**GAPU71B**	**EMO90**	**EMO90**	**DCA18**	**DCA18**	**DCA15**	**DCA15**	**TREATMENT**	**LIKELY FATHER**	**LOD SCORE**

No	DCA5	DCA5	EMO3	EMO3	DCA3	DCA3	GAPU101	GAPU101	GAPU71B	GAPU71B	EMO90	EMO90	DCA18	DCA18	DCA15	DCA15	TREATMENT	LIKELY FATHER	LOD SCORE
158	204	204	211	211	236	240	194	218	124	130	186	186	169	177	243	263	x Koroneiki R3	Tosca	below 0.89
159	200	204	215	219	240	246	186	186	124	126	186	186	175	177	254	263	x Koroneiki R4	Arbequina	below 0.89
162	204	204	206	215	236	242	192	218	130	144	186	186	169	171	243	254	x Koroneiki R4	Tosca	below 0.89
**No**	**DCA5**	**DCA5**	**EMO3**	**EMO3**	**DCA3**	**DCA3**	**GAPU101**	**GAPU101**	**GAPU71B**	**GAPU71B**	**EMO90**	**EMO90**	**DCA18**	**DCA18**	**DCA15**	**DCA15**	**TREATMENT**	**LIKELY FATHER**	**LOD SCORE**
